# Effect of Xiaoyaosan on brain volume and microstructure diffusion changes to exert antidepressant-like effects in mice with chronic social defeat stress

**DOI:** 10.3389/fpsyt.2024.1414295

**Published:** 2024-09-19

**Authors:** Yongxin Li

**Affiliations:** Guangzhou Key Laboratory of Formula-pattern Research Center, School of Traditional Chinese Medicine, Jinan University, Guangzhou, China

**Keywords:** antidepressant-like effect, XYS, fractional anisotropy, brain volume, CSDS model mouse

## Abstract

**Objective:**

Depression is a prevalent mental disorder characterized by persistent negative mood and loss of pleasure. Although there are various treatment modalities available for depression, the rates of response and remission remain low. Xiaoyaosan (XYS), a traditional Chinese herbal formula with a long history of use in treating depression, has shown promising effects. However, the underlying mechanism of its therapeutic action remains elusive. The aim of this study is to investigate the neuroimaging changes in the brain associated with the antidepressant-like effects of XYS.

**Methods:**

Here, we combined voxel-based morphometry of T2-weighted images and voxel-based analysis on diffusion tensor images to evaluate alterations in brain morphometry and microstructure between chronic social defeat stress (CSDS) model mice and control mice. Additionally, we examined the effect of XYS treatment on structural disruptions in the brains of XYS-treated mice. Furthermore, we explored the therapeutic effect of 18β-glycyrrhetinic acid (18β-GA), which was identified as the primary compound present in the brain following administration of XYS. Significant differences in brain structure were utilized as classification features for distinguishing mice with depression model form the controls using a machine learning method.

**Results:**

Significant changes in brain volume and diffusion metrics were observed in the CSDS model mice, primarily concentrated in the nucleus accumbens (ACB), primary somatosensory area (SSP), thalamus (TH), hypothalamus (HY), basomedical amygdala nucleus (BMA), caudoputamen (CP), and retrosplenial area (RSP). However, both XYS and 18β-GA treatment prevented disruptions in brain volume and diffusion metrics in certain regions, including bilateral HY, right SSP, right ACB, bilateral CP, and left TH. The classification models based on each type of neuroimaging feature achieved high accuracy levels (gray matter volume: 76.39%, AUC=0.83; white matter volume: 76.39%, AUC=0.92; fractional anisotropy: 82.64%, AUC=0.9; radial diffusivity: 76.39%, AUC=0.82). Among these machine learning analyses, the right ACB, right HY, and right CP were identified as the most important brain regions for classification purposes.

**Conclusion:**

These findings suggested that XYS can prevent abnormal changes in brain volume and microstructure within TH, SSP, ACB, and CP to exert prophylactic antidepressant-like effects in CSDS model mice. The neuroimaging features within these regions demonstrate excellent performance for classifying CSDS model mice from controls while providing valuable insights into the antidepressant effects of XYS.

## 1 Introduction

Depression is a globally prevalent mental disorder characterized by abnormal changes in several cognitive domains, including attention, memory, and emotion processing (1). In addition, depression is the primary cause of persistent impairment in quality of life (2). Currently, depression disorder is recognized as a significant public health issue with a global burden (3). Various pharmacological treatments are available for depression in clinical practice. However, these antidepressant medications exhibit low response rates and significant drug side effects. Less than half of patients achieve remission after the initial pharmacological treatment. The current severe state of depression necessitates the development of more efficient antidepressant drugs.

An increasing number of studies has demonstrated the enduring antidepressant effects and minimal adverse reactions associated with this traditional Chinese medicine in the treatment of depression (4–7). Xiaoyaosan (XYS), a traditional Chinese herbal formula used alone or in combination with antidepressants, has been employed for treating depression in China (4). Several preclinical studies have indicated that XYS can ameliorate core symptoms of behavioral despair and anhedonia in model rats with chronic unpredictable mild stress (8–10). In clinical research, the use of XYS alone or combined with Western medicines has been shown to alleviate depression symptoms (5, 11). When combined with Western medicines, XYS demonstrates a significant comprehensive effect on the treatment of depression: superior Hamilton Depression Rating Scale and Self-Rating Depression Scale scores (12). Overall, these preclinical and clinical studies have demonstrated the safety and efficacy of XYS in the treatment of depression. In order to understand the mechanisms of the effects of XYS in depression, previous studies have primarily focused on pharmacological mechanisms and provided many theories, such as monoamine neurotransmitters, synaptic plasticity, inflammatory response, neuroprotection, and brain–gut axis (6). However, the previous studies into the mechanisms of the effects of XYS have been too broad.

With advancements in magnetic resonance imaging (MRI), neuroimaging techniques have become widely utilized for exploring the brain mechanisms associated with psychiatric disorders, such as depression (13, 14). Neuroimaging studies have revealed differences in brain volume, function, and connectivity among individuals with depression within regions involved in emotion processing and mood regulation (14–16). Recent meta-analysis has identified specific cortical and subcortical areas implicated in major depression disorder through alterations in brain volume and activity including the prefrontal regions, the cingulate cortex, the hippocampus, the insula, the thalamus, and the amygdala (17–19). Furthermore, MRI technology has been employed to investigate the impact of antidepressant treatments on both structural changes and functional alterations within patients with depressive disorder (20). For instance, a previous study observed an increase in left amygdala volume following six subanesthetic dose infusions of ketamine treatments that corresponded to an improvement in depressive symptoms (21). A previous study investigating the impact of ketamine on white matter (WM) microstructure revealed significant alterations in neurite microstructural features within occipitotemporal tracts, as well as changes in neurite density within tracts connecting the basal ganglia, thalamus, and cortex that were associated with improvements in anhedonia (22). Antidepressant treatments such as ketamine and fluoxetine have been found to influence brain function and structure (22, 23). These findings collectively demonstrate the potential of the MRI technique to elucidate functional pathways and systems underlying therapeutic effects at a macro scale.

By considering XYS’s potential antidepressant properties, the neuroimaging technique can also aid in identifying functional and structural changes contributing to its therapeutic effects. This perspective has been supported by previous studies conducted using a chronic unpredictable mild stress mouse model of depression (24, 25), which observed significant upregulation of blood oxygen level-dependent signals in the limbic system of a model mouse, with modified XYS normalizing brain signals to exert antidepressant effects. Although XYS therapeutic effects on brain functional activity have been studied on the model mouse, few studies have shown sufficient evidence in exploring the antidepression effects of XYS through brain macro- or microstructural plasticity. Currently, various imaging analysis approaches have emerged. Voxel-based morphometry (VBM) is a practical structural analysis method, which provides a comprehensive assessment of anatomic structure throughout the entire brain (26). Gray matter (GM) and WM volumes are commonly used indicators to represent the volume of specific gray matter/white matter areas. Diffusion tensor image (DTI) is a powerful method for investigating brain microstructural abnormalities and is widely employed as a non-invasive tool for detecting changes in depression (22, 27). However, the impact of XYS on GM structure and WM architecture across the whole brain remains largely unknown. Utilizing these neuroimaging approaches in depression research would provide valuable insights into understanding the antidepressant mechanism of XYS.

Based on previous research evidence in depression, we hypothesized that XYS could reduce depression by preventing disruptions in both brain morphometry and microstructure aspects. The convergence findings regarding XYS’s antidepressive effects might be localized in regions such as the thalamus, amygdala, and cortical cortex. To test this hypothesis, we employed a chronic social defeat stress (CSDS) model, which has been proposed as an animal model of depression (28, 29). Approximately 70% of mice were found to be susceptible after exposure to social defeat stress (28). We chose this CSDS model because it better simulates typical human depression caused by excessive social pressure and frequent frustration during daily social activities. Additionally, this study serves as an extension of our previous investigations into studying the mechanisms underlying depression in animals (30). We utilized VBM of T2-weighted images and voxel-based analysis of DTI to assess changes in brain morphometry and microstructure between CSDS model mice and controls. Furthermore, we investigated the treatment effect of XYS on brain structure disruption in XYS-treated mice. The potential therapeutic impact of 18β-glycyrrhetinic acid (18β-GA), identified as the primary compound present in the brain following XYS injection (30), was also explored. In recent years, there has been growing interest in employing machine learning techniques for analyzing neuroimaging data (31). In this study, significant differences observed in brain structure were selected as classification features for subsequent machine learning analyses aimed at determining whether these structural differences can correctly distinguish mice with a depression phenotype form control mouse.

## 2 Methods

### 2.1 Animals

The animal studies conducted in this research were approved by the National Institutional Animal Care and Ethical Committee of Jinan University (Approval No. IACUC-20230912-02). All methodologies employed in this study strictly adhered to the approved guidelines and regulations.

Eight-week-old male adult C57BL/6J (body weight 20 g–25 g) mice were purchased from Guangdong Experimental Animal Center, China. All animals were housed in an accredited animal care facility under controlled temperature and humidity conditions, following a 12-h light/dark cycle (lights on between 07:00 and 19:00). The animals were housed with ad libitum access to food and water. The animals in this study were randomly allocated to experimental groups with age match. Random allocation was performed for age matching within the experimental groups consisting of 33 C57BL/6J mice, comprising 8 control group, 9 CSDS model group, 8 CSD model + XYS-treated group, and 8 CSDS model + 18β-GA-treated group. Among these groups, the CSDS group, XYS-treated group, and 18β-GA treated group were given CSDS program for 10 days.

### 2.2 Preparation of drugs

XYS is a herbal formulation consisting of eight Chinese herbs: Angelica sinensis Diels (AS); Paeonia lactiflora Pallas (PN); Bupleurum chinense DC. (BR); Atractylodes macrocephala (AMR); Poria cocos (Schweinitz) Wolf (PR); Zingiber officinale Roscoe (ZRR); Glycyrrhiza uralensis Fischer (GRH); Mentha canadensis Linnaeus (MH). The XYS used in this study was provided by Jiuzhitang Co., Ltd. (Changsha, China), and produced according to the production technology specified in the 2020 edition of the Chinese Pharmacopoeia, as regulated by the State Pharmacopoeia Commission (32). One gram of powder was obtained from every 2.10 g of the raw herbs for administration purposes. XYS was orally administered to mice at a dosage of 0.125 g/kg, 30 min before CSDS induction, for a duration of 10 days. For comparison, an additional treatment group received intraperitoneal administration of 18β-GA (catalog number: M17224, Meryer, Shanghai, China) dissolved in a solution containing 10% corn oil and physiological saline at a dosage of 10 mg/kg, also given prior to chronic social defeat stress induction for 10 consecutive days.

### 2.3 Chronic social defeat stress experiment

For the CSDS depression model, the C57BL/6 mice were exposed to different CD1 mice for 10 min for a total of 10 days. During this period, both CD1 mice and C57BL/6 mice were housed in separate halves of cages divided by a perforated Plexiglas divider that allowed visual, olfactory, and auditory contact between them within the first 24 h following each social defeat session. After completion of the final session of social defeat stress exposure, C57BL/6 mice were individually housed separately from other animals involved in the experiment. The social interaction test was performed to examine the mice that were susceptible and unsusceptible to social defeat stress. The entire experimental process of the CSDS model follows exactly as described in our recent article (30).

For the social interaction test, an open box (42 × 42 cm) was used, which has an interaction zone including a mesh-plastic target box (10 × 4.5 cm) and two opposing corner zones. This test was divided into two parts: no social target and social target. In the no social target part, the test mouse was placed into an open field arena for 2.5 min with no social target (no CD1 mouse) in the mesh-plastic target box. After this initial phase, the mouse was placed into an open field arena again in the second 2.5 min with a social target (a novel CD1 mouse) in the mesh-plastic target box. The residence time in the interaction zone was counted by using the stopwatch, the time of ratio for social target, and no social target was calculated. All mice used in this study were successfully modeled except for those in the normal group, and our research builds upon our previously published work (30). Mice that did not meet modeling criteria were excluded from analysis.

### 2.4 MRI acquisition

The magnetic resonance imaging was acquired using a Bruker 9.4T scanner (Bruker BioSpin Ettlingen, Germany). T2-weighted images and DTI were performed on all mice. During the scan, an animal monitoring unit (Small Animal Instruments Inc., New York, NY, USA) was utilized to record the heart rate and respiratory frequency of the mice. To maintain normal body temperature throughout the experiment, a hot water circulation system (medres, Cologne, Germany) was employed to ensure physiological stability. Initially, mice were anaesthetized with a mixture of 1% oxygen and 3% isoflurane and then placed on an MRI-compatible stand with a mixture of 0.5% oxygen and 2.5% isoflurane during data acquisition. To minimize head movement, the mice were fixed on an animal bed using a MRI-compatible cradle with surface coil and body coil.

The acquisition parameters for T2-weighted images were as follows: TurboRARE sequence with TR/TE = 2,500/33 ms, slice thickness = 0.7 mm, average = 2, matrix size = 256 × 256 × 17, spatial resolution = 0.08 × 0.08 × 1 mm^3^, flip angle = 180°. Diffusion tensor image (DTI) data were acquired using an echo-planar imaging sequence. The main imaging parameters were TR = 3,000 ms, TE = 20.7 ms, slice thickness = 1 mm, matrix size = 108 × 96 × 17, spatial resolution = 0.19 × 0.21 × 1 mm^3^, flip angle = 90°, average = 1, 5 b-value = 0 (b_0_) images, followed by 30 diffusion encoding gradient directions with a diffusion weighting (b) of 660 s/mm^2^.

**Figure 1:**
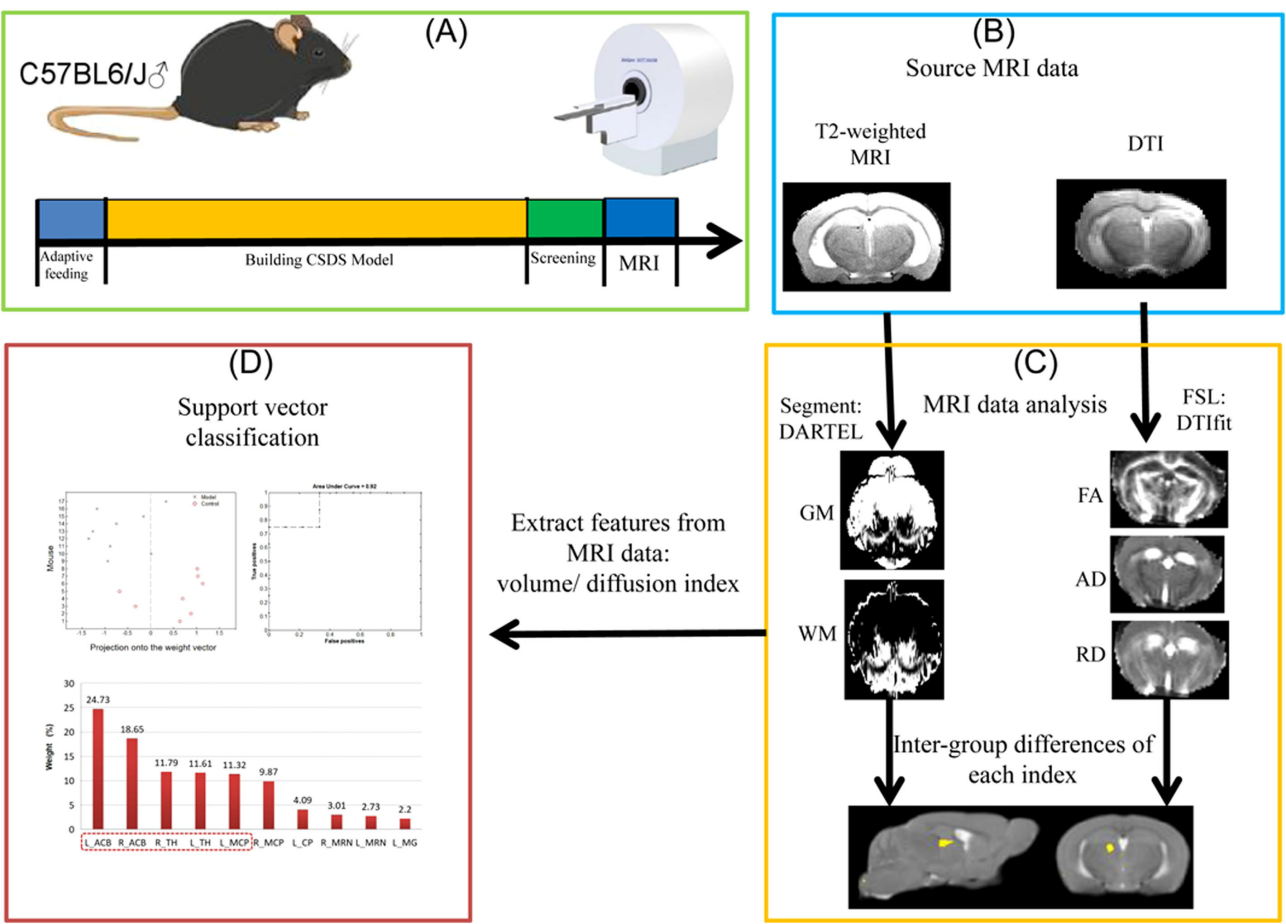
Flowchart of the neuroimage data. The figure illustrates the processing steps of MRI data. **(A)** CSDS model construction and data collection process. **(B)** Type of MRI data scanning of mouse brain. **(C)** Specific methods and procedures of mouse brain MRI data. **(D)** Using machine learn method for classification.

The flowchart summarizing the neuroimage data can be found in **Figure 1**.

### 2.5 Imaging data processing

The Bruker raw data were converted to the Neuroimaging Informatics Technology Initiative (NIfTi) format using MRIcron software for imaging data processing.

#### 2.5.1 Anatomical MRI data analysis

T2-weighted images of the brain were carried out using the voxel-based morphometry (VBM) tool of the SPM8 software package (SPM, https://www.fil.ion.ucl.ac.uk/spm). (1) The image orientation of the T2-weighted data was adjusted to match the common three-dimensional coordinate system with three planes: L = left-to-right, I = inferior-to-superior, P = posterior-to-anterior. This adjustment was performed using the 3drefit command in AFNI software (https://afni.nimh.nih.gov/). (2) To enable SPM to process the data with human-like voxel sizes, a scaling factor of 10 was applied after brain extraction using AFNI software. (3) The scaled and masked brain images were then normalized by registering them to the MSA mouse template in SPMmouse (https://www.fil.ion.ucl.ac.uk/spm/ext/#SPMMouse). This step facilitated further analysis. (4) Normalized T2-weighted images were segmented into gray matter (GM), white matter (WM), and cerebrospinal fluid (CSF), following a non-linear deformation field estimation that aligned mouse tissue probability maps provided by SPMMouse with each individual mouse image (33). (5) Segmented GM and WM images underwent non-linear transformation using Diffeomorphic Anatomical Registration Through Exponentiated Lie Algebra (DARTEL) techniques followed by modulation to create a modified template, which was subsequently smoothed with a 4-mm full-width at half maximum isotropic Gaussian kernel. (6) Statistical analysis comparing volume differences between groups was conducted on observed GM and WM images using the SPM8 statistical software package.

#### 2.5.2 Diffusion MRI data analysis

For DTI data, b0 images were extracted with the skull intact. Non-brain tissue was removed by applying a binary mask of brain extraction to both the b0 and the original DTI data. The data dimension was scaled by a factor of 10 to stimulate human-like voxel sizes. Post-processing of DTI was performed using FMRIB software library version 6.0.2 (FSL, created by the Analysis Group, Oxford, UK). Distortions and motion artifacts were corrected using FSL’s eddy correct (34). Tensors were fitted using the b-factor and diffusion direction matrix with the DTIfit toolbox to calculate eigenvalues (λ1, λ2, and λ3), resulting eigenvectors fractional anisotropy (FA) indices for each voxel in diffusion-weighted brain maps including whole-brain FA maps, radial diffusivity (RD) as the mean of the second and third eigenvalues ((λ1 + λ2)/2), and axial diffusivity (AD) as the principal diffusion eigenvalue (λ1). Each mouse’s b0 brain images were coregistered to a customized reference brain template (DARTEL template of T2-weighted data), and then a transforming matrix was applied to register respective DTI index maps. Normalized diffusion index maps smoothed using an isotropic Gaussian filter with full-width at half maximum of 4 mm.

### 2.6 Statistical analyses

For both the smoothed GM and WM maps, a two-tailed, two-sample t-test was conducted using a linear model in SPM to compare brain volume information between the control and model groups at the voxel-wise level. The aim of this step was to identify regions exhibiting whole-brain morphological changes in mice with depression. Statistical significance of GM and WM volume changes was set at an uncorrected voxel-level height threshold of p < 0.005, with a cluster extent threshold of 40 voxels. Regions that survived this correction were considered significantly different and selected as regions of interest (ROIs). The GM and WM volumes of each ROI were extracted for all mice. To compare discrepancies among the four groups, statistical analysis was performed on the volume data from these ROIs using one-way ANOVA in SPSS 20.0, followed by *post hoc* tests. A significance level of p < 0.05 (n = 33) was considered statistically significant. Based on this analysis, we observed the antidepressive-like effects of XYS treatment on brain anatomy.

The smoothed FA maps underwent voxel-wise statistics based on spatial analysis, employing two-tailed, two-sample t-tests to evaluate microstructural alterations between the control and model groups. A significance threshold of p < 0.005 was applied to identify regions exhibiting statistical significance in the FA maps. These significant regions were selected, and the mean FA values within each region were extracted of all mice. Subsequently, changes in FA among the four groups were assessed to investigate the antidepressive effects of XYS treatment on brain microstructure. One-way ANOVA was conducted on the average FA values obtained from these ROIs across the four groups. *Post-hoc* analyses were performed when significant differences were observed. p < 0.05 was considered statistically significant. Similar analyses were performed for other diffusion indices (AD and RD) to further elucidate the underlying mechanisms driving diffusion changes.

### 2.7 Support vector machine analysis

The support vector machine (SVM) method was implemented using PRoNTo (Pattern Recognition for Neuroimaging Toolbox) software version 2.1 (http://www.mlnl.cs.ucl.ac.uk/pronto) in MATLAB. This approach aimed to assess the discriminative capability of neuroimaging values extracted from abnormal brain regions in distinguishing depress-model mice from control mice. Regions exhibiting significant difference between the model and control groups were selected based on the aforementioned neuroimaging analyses, and their corresponding neuroimaging values were employed as features for SVM classification. A “leave-one-out” cross-validation strategy was adopted during this process. Permutation tests were conducted to estimate the statistical significance of the classification accuracy (35), involving repeated execution of the classification procedure for 1,000 iterations. The number of permutations achieving higher sensitivity and specificity than the true labels was used to derive a p value. Statistical significance of classification accuracy was determined by this process. Additionally, weight maps summarizing the contributions of each ROI toward the linear predictive function were computed to enhance interpretability.

## 3 Results

### 3.1 Prophylactic effect of XYS and 18β-GA on depression-like behaviors in the CSDS model

This study is a continuation of our recently published research (30), where the behavioral data have already been reported. To avoid redundant publication, this study only includes brief descriptions of the main outcome measures to demonstrate the success of the model and the preventive effects of XYS and 18β-GA. In the no-target social interaction test (SIT), there were no significant differences in social interaction duration among groups. However, in the target SIT, both XYS and 18β-GA increased social interaction time in CSDS mice. No significant changes were observed in locomotion tests across all four groups. Both XYS and 18β-GA significantly reduced immobility time in forced swimming tests for CSDS mice. Additionally, both XYS and 18β-GA significantly ameliorated decreased sucrose preference observed in CSDS mice during the 1% sucrose preference test. Detailed behavioral outcomes can be found in our recently published article (30).

### 3.2 Chronic stress decreased the brain GM and WM volumes at the whole-brain level of mice

**Figure 2:**
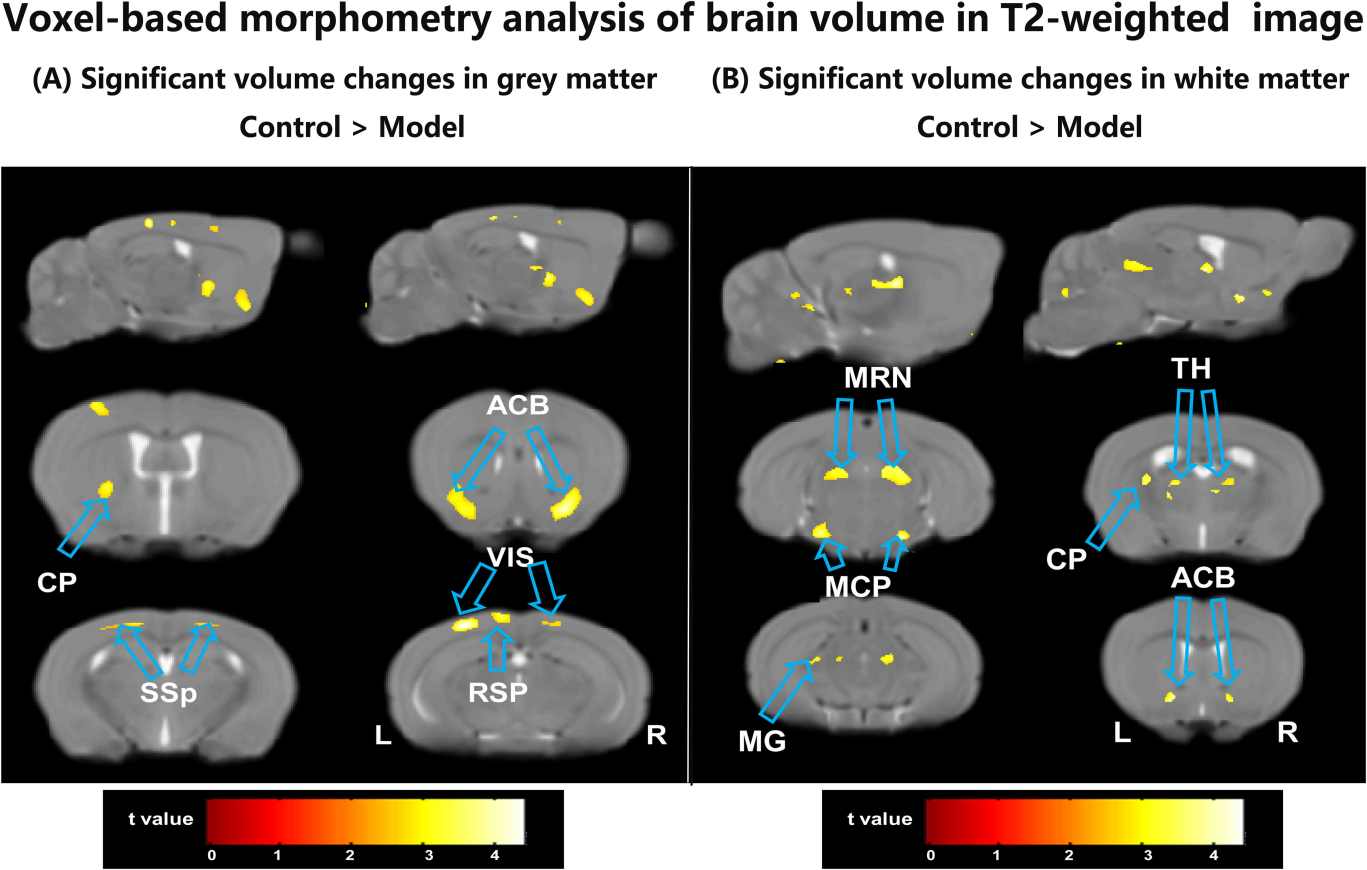
The differences between the model group and the control group by VBM analysis of the T2-weighted structure images. **(A)** The regions showed a significant decrease of the GM volumes in the model mice compared with the control mice. **(B)** The regions showed a significant decrease of the WM volumes in the model mice compared with the control mice. ACB, nucleus accumbens; CP, caudoputamen; MCP, middle cerebellar peduncle; MG, medial geniculate complex of thalamus; MRN, midbrain reticular nucleus; RSP, retrosplenial area; SSP, primary somatosensory area; TH, thalamus; VIS, retrolateral lateral visual area; L, left; R, right.

**Figure 3:**
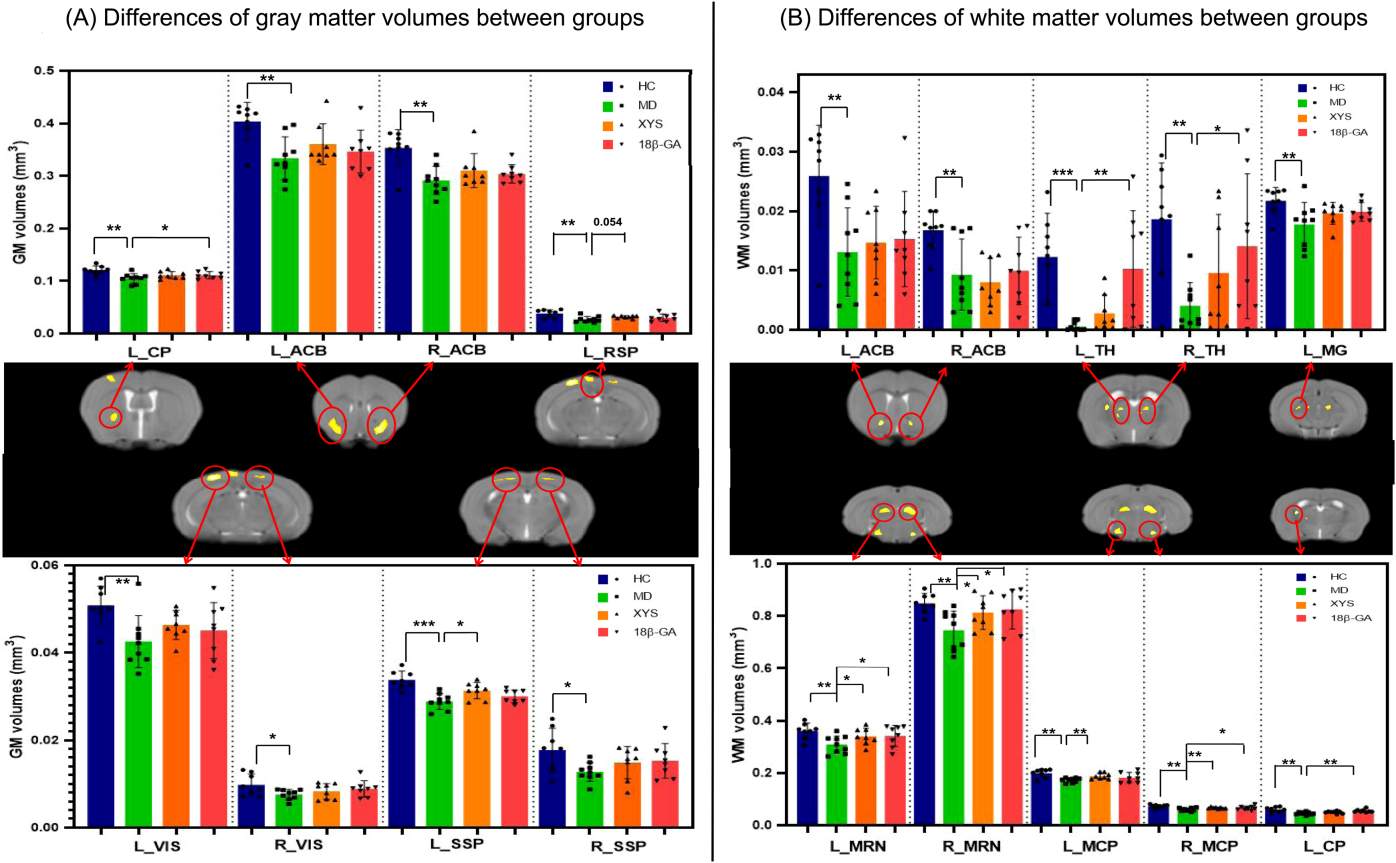
Effects of the XYS treatment on brain anatomic volumes. **(A)** Significant differences of the GM volumes in each brain region of interest between groups. **(B)** Significant differences of the WM volumes in each brain regions of interest between groups. Post t-test: *p<0.05, **p<0.01, ***p<0.001.

The GM results from VBM analysis are presented in **Figure 2A**. Compared with the control group, the CSDS model group exhibited a lower GM volume primarily localized mainly in the bilateral primary somatosensory area (SSP), bilateral retrolateral lateral visual area (VIS), bilateral nucleus accumbens (ACB), left caudoputamen (CP) and left retrosplenial area (RSP) as shown in **Table 1**. These regions displaying significant GM volume abnormalities were selected as ROIs, and the GM volume values were extracted from each ROI of all mice. Although most ROIs in both treated groups showed an enhancement compared with the model group, this enhancement did not reach statistical significance, as depicted in **Figure 3A**. Compared with the model group, the GM volumes of left SSP in the XYS-treated group showed a significant increase (t = 2.73, p = 0.015) and the GM volumes of left CP in the 18β-GA-treated group also showed a significant increase (t = 2.58, p = 0.022).

**Table 1:** ANOVA test results among groups.

Image indicators	ROIs	F-test		*Post-hoc* test					
				Control vs. model		XYS vs. model		18β-GA vs. model	
		F value	p value	t value	p value	t value	p value	t value	p value
GM volume	L caudoputamen	5.724	0.003	3.749	0.002	1.511	0.152	**2.17**	**0.048**
	L nucleus accumbens	4.967	0.007	3.725	0.002	1.383	0.187	0.66	0.52
	R nucleus accumbens	7.230	0.001	4.118	0.001	1.292	0.216	1.113	0.283
	L retrosplenial area	5.474	0.004	3.657	0.002	2.092	0.054	0.996	0.335
	L retrolateral lateral visual area	3.763	0.021	3.236	0.006	1.6	0.13	0.847	0.41
	R retrolateral lateral visual area	2.292	0.097	2.455	0.027	1.0	0.33	1.769	0.097
	L primary somatosensory area	11.459	0.000	5.294	0.000	**2.73**	**0.015**	1.46	0.165
	R primary somatosensory area	2.479	0.081	2.752	0.015	1.466	0.163	1.652	0.119
WM volume	L nucleus accumbens	4.884	0.007	3.309	0.005	0.475	0.642	0.591	0.564
	R nucleus accumbens	5.124	0.006	3.137	0.007	0.496	0.627	0.223	0.826
	L thalamus	6.928	0.001	4.767	0.000	2.089	0.054	**2.965**	**0.01**
	R thalamus	3.447	0.029	3.948	0.001	1.396	0.183	**2.249**	**0.04**
	L caudoputamen	7.916	0.001	4.00	0.001	1.637	0.122	**3.439**	**0.004**
	L medial geniculate complex of thalamus	3.477	0.029	2.623	0.019	1.276	0.221	1.524	0.148
	L midbrain reticular nucleus	3.370	0.032	3.326	0.005	**2.453**	**0.028**	**2.226**	**0.043**
	R midbrain reticular nucleus	4.076	0.016	3.573	0.003	**2.379**	**0.032**	**2.556**	**0.023**
	L middle cerebellar peduncle	4.896	0.007	4.318	0.001	**3.109**	**0.007**	1.269	0.224
	R middle cerebellar peduncle	6.555	0.002	4.280	0.001	**3.328**	**0.005**	**2.192**	**0.047**
FA value	R hypothalamus	4.437	0.011	3.934	0.001	**2.17**	**0.048**	**3.55**	**0.003**
	R hypothalamus	7.018	0.001	3.456	0.004	**2.706**	**0.017**	**8.295**	**0.000**
	R hypothalamus	5.353	0.005	4.074	0.001	**2.339**	**0.035**	**2.613**	**0.02**
	R basomedial amygdala nucleus	6.887	0.001	4.337	0.001	0.911	0.378	1.61	0.13
	L basomedial amygdala nucleus	6.208	0.002	3.845	0.002	1.531	0.147	1.729	0.104
	R primary somatosensory area	5.989	0.003	4.492	0.001	**3.922**	**0.001**	**2.64**	**0.019**
	R nucleus accumbens	6.205	0.002	3.663	0.003	**3.421**	**0.004**	**2.52**	**0.023**
	R caudoputamen	4.620	0.009	3.401	0.004	**2.34**	**0.034**	0.654	0.523
AD value	L thalamus	3.611	0.025	3.138	0.007	1.275	0.222	**2.815**	**0.013**
	L hypothalamus	4.226	0.014	2.484	0.026	1.504	0.153	**4.609**	**0.000**
	R posterior hypothalamus	4.763	0.008	3.439	0.004	1.469	0.163	1.522	0.149
	R caudoputamen	5.018	0.006	4.569	0.000	**2.142**	**0.049**	0.369	0.717
	lateral septal nucleus	14.523	0.000	7.372	0.000	**3.084**	**0.008**	**2.234**	**0.042**
	L caudoputamen	8.645	0.000	4.938	0.000	**2.371**	**0.033**	1.568	0.139
	R caudoputamen	4.191	0.014	3.05	0.009	**2.399**	**0.031**	1.742	0.103
	R nucleus accumbens	5.184	0.005	3.905	0.002	**2.237**	**0.042**	1.8	0.094
RD value	L thalamus	5.611	0.004	2.26	0.04	**2.394**	**0.031**	**2.525**	**0.024**

The data with bold type indicated significant differences between the two intervention groups and the model group.

**Figure 2B** illustrates the WM results obtained from VBM analysis. In comparison with the control group, the untreated model group displayed a lower WM volume localized mainly localized in left CP, bilateral ACB, bilateral thalamus (TH), bilateral midbrain reticular nucleus (MRN), bilateral middle cerebellar peduncle (MCP), and left Medial geniculate complex of thalamus (MG) (seen in **Table 1**). These regions exhibiting significant WM volume abnormalities were chosen as ROIs for extracting the WM volume from each ROI of all mice involved in this study. When comparing with the CSDS model group, both treated groups demonstrated a significant increase in WM volumes of bilateral MRN (XYS-treated: L MRN t = 2.45, p = 0.028, R MRN: t = 2.38, p = 0.032; 18β-GA-treated: L MRN t = 2.23, p = 0.043, R MRN: t = 2.56, p = 0.023) and right MCP (XYS-treated: t = 3.33, p = 0.005; 18β-GA treated: t = 2.10, p = 0.05) in showed significant increase (**Figure 3B**). Additionally, the WM volumes of bilateral TH (L TH: t = 2.97, p = 0.01, R TH: t = 2.25, p = 0.04) and left CP (t = 3.44, p = 0.004) in the 18β-GA-treated group showed a significant increase compared with the CSDS model group (**Figure 3B**). In the XYS-treated group, there was an observed increase in white matter (WM) volumes in these regions; however, statistical significance was not achieved. When compared with the CSDS model group, a significant elevation in WM volumes of the left MCP was detected in the XYS-treated group showing a significant increase (t = 3.11, p = 0.007), which were not observed in the 18β-GA-treated group.

### 3.3 XYS significantly increased the WM diffusion properties in CSDS mice

**Figure 4:**
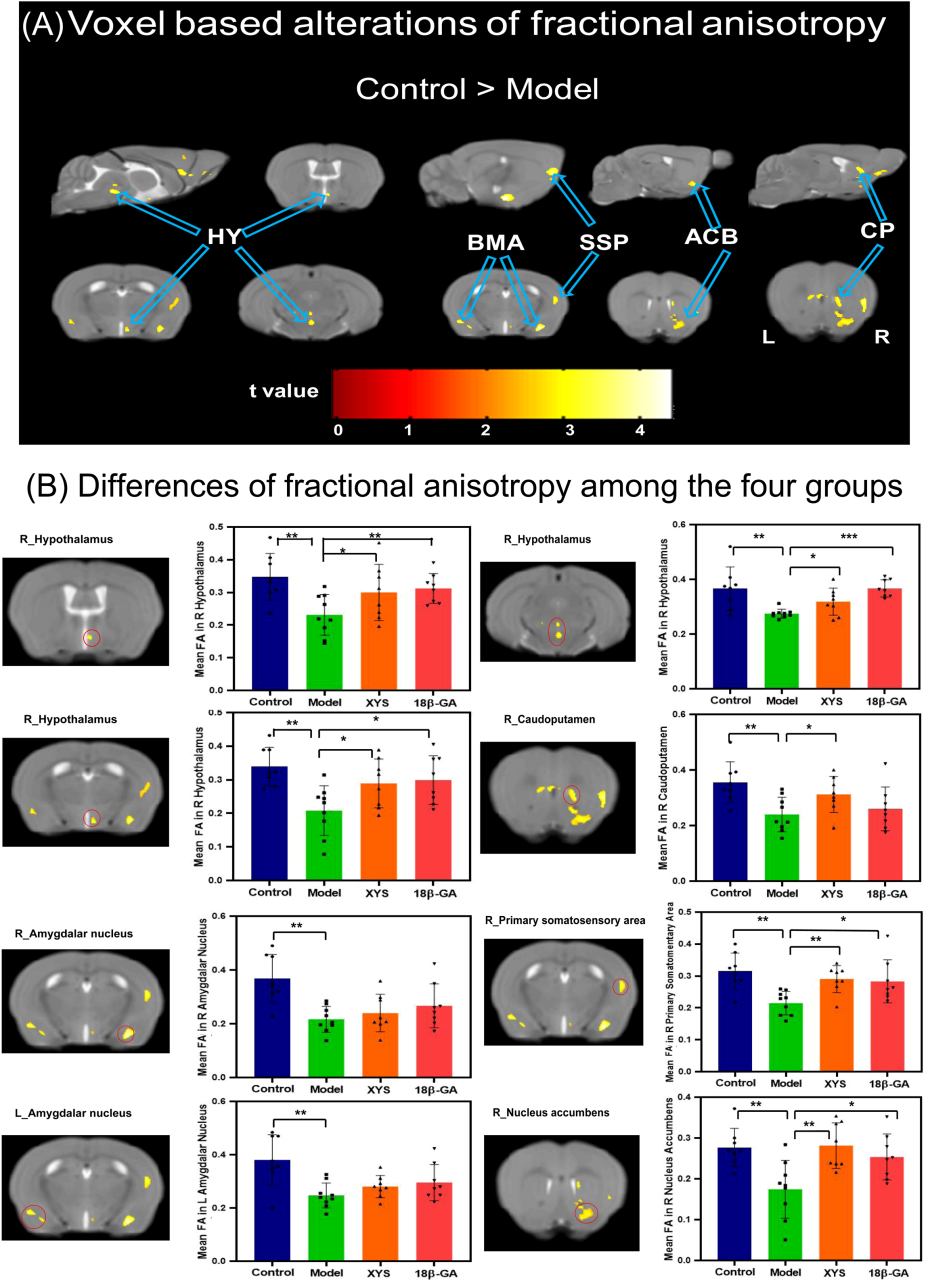
From FA diffusion maps, WM microstructural differences detected among the four groups. **(A)** Model mice showed a significant decrease of FA compared with the control mice by voxel-based statistics. **(B)** Effect of the XYS treatment on brain diffusion properties of FA in each brain of interest between groups. ACB, nucleus accumbens; BMA, basomedial amygdalar nucleus; CP, caudoputamen; HY, hypothalamus; SSP, primary somatosensory area; L, left; R, right. Post t-test: *p<0.05, **p<0.01, ***p<0.001.

We also performed whole-brain voxel-based DTI analysis to validate the impact of social defeat stress on brain diffusion properties in depression-like mice. As depicted in **Figure 4A**, significant reductions in FA values were observed in several regions of the CSDS model group compared with the control group. Notably, lower FA was predominantly found mainly in the right hypothalamus (HY), bilateral basomedial amygdala nucleus (BMA), right CP, right ACB, and right SSP (as shown in **Table 1**). These regions exhibiting significant intergroup differences were selected as our ROIs, and the mean FA values were extracted from these ROIs for all mice (**Figure 4B**). In comparison with the CSDS model group, both treatment groups exhibited a significant increase in mean FA values within the right HY (XYS-treated: HY_1, t = 2.17, p = 0.048, HY_2, t = 2.71, p = 0.017, HY_3, t = 2.34, p = 0.035; 18β-GA-treated: HY1, t = 3.55, p = 0.003, HY2, t = 8.20, p = 0.000, HY3, t = 2.61, p = 0.02), right SSP (XYS-treated: t = 4.15, p = 0.001; 18β-GA-treated: t = 2.75, p = 0.016), and right ACB (XYS-treated: t = 3.603, p = 0.003; 18β-GA-treated: t = 2.723, p = 0.016). The XYS-treated group also showed a significant increase in mean FA values within the right CP compared with the CSDS model group (t = 2.311, p = 0.037). Although an increase was observed for other regions of interest within both treatment groups when compared with the CSDS model group, the difference did not reach statistical significance.

**Figure 5:**
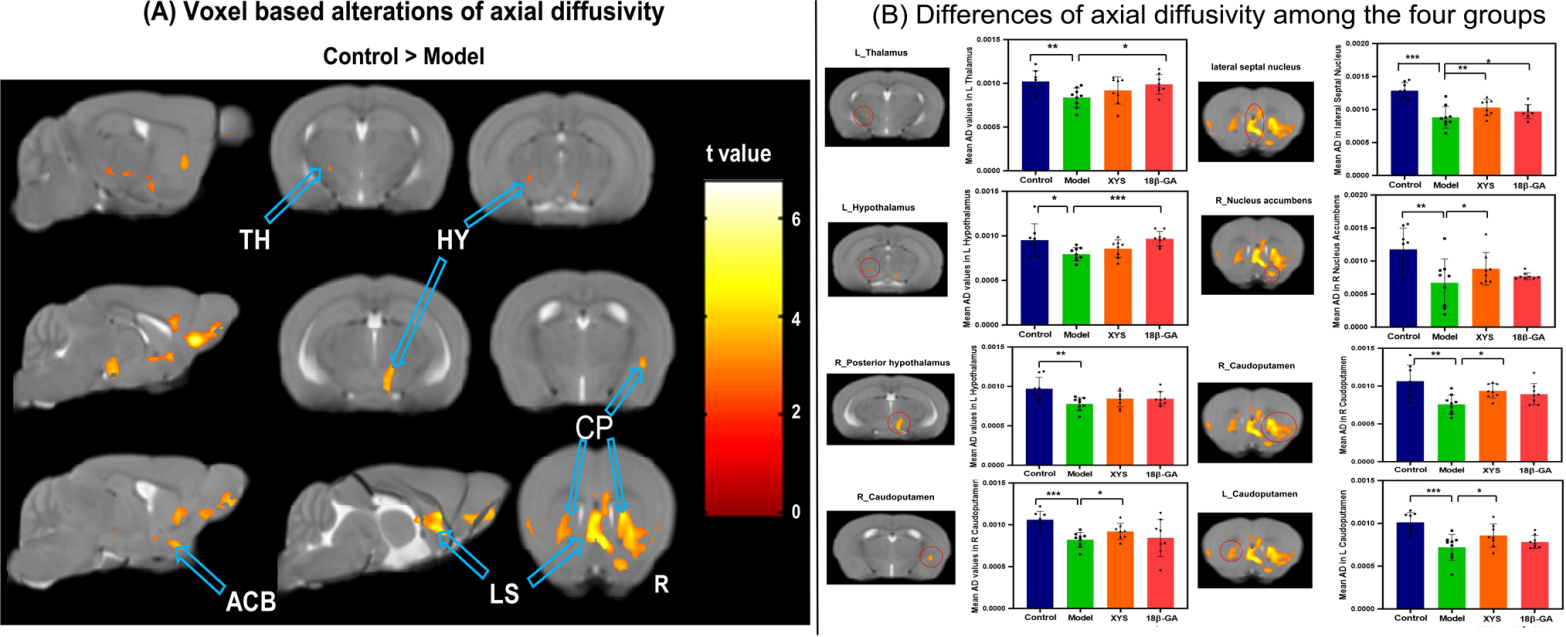
From AD maps, WM microstructural differences were detected among the four groups. **(A)** Model mice showed significant decrease of AD compared with the control mice by voxel-based statistics. **(B)** Effect of the XYS-treatment on brain diffusion properties of AD in each brain of interest between groups. ACB, nucleus accumbens; CP, caudoputamen; HY, hypothalamus; LS, lateral septal nucleus; TH, thalamus; L, left; R, right. Post t-test: *p<0.05, **p<0.01, ***p<0.001.

The voxel-based analyses of the AD maps were conducted, and the main findings are presented in **Figure 5A**. In comparison with the control group, the CSDS model group exhibited lower AD values in the left TH, bilateral HY, bilateral CP, right ACB, and lateral septal nucleus (LS) (seen in **Table 1**). The mean AD values within these regions were extracted from all mice. Notably, compared with the CSDS model group, a significant increase in AD values was observed in the left TH (t = 2.69, p = 0.018), left HY (t = 5.01, p = 0.000), and LS (t = 2.234, p = 0.042) following treatment with 18β-GA. Similarly, when compared with the CSDS model group, administration of XYS resulted in a significant increase in AD values within the right ACB (t = 2.24, p = 0.042), bilateral CP (L: t = 2.37, p = 0.033, R: t = 2.40, p = 0.031), and LS (t = 3.08, p = 0.008) (**Figure 5B**).

**Figure 6:**
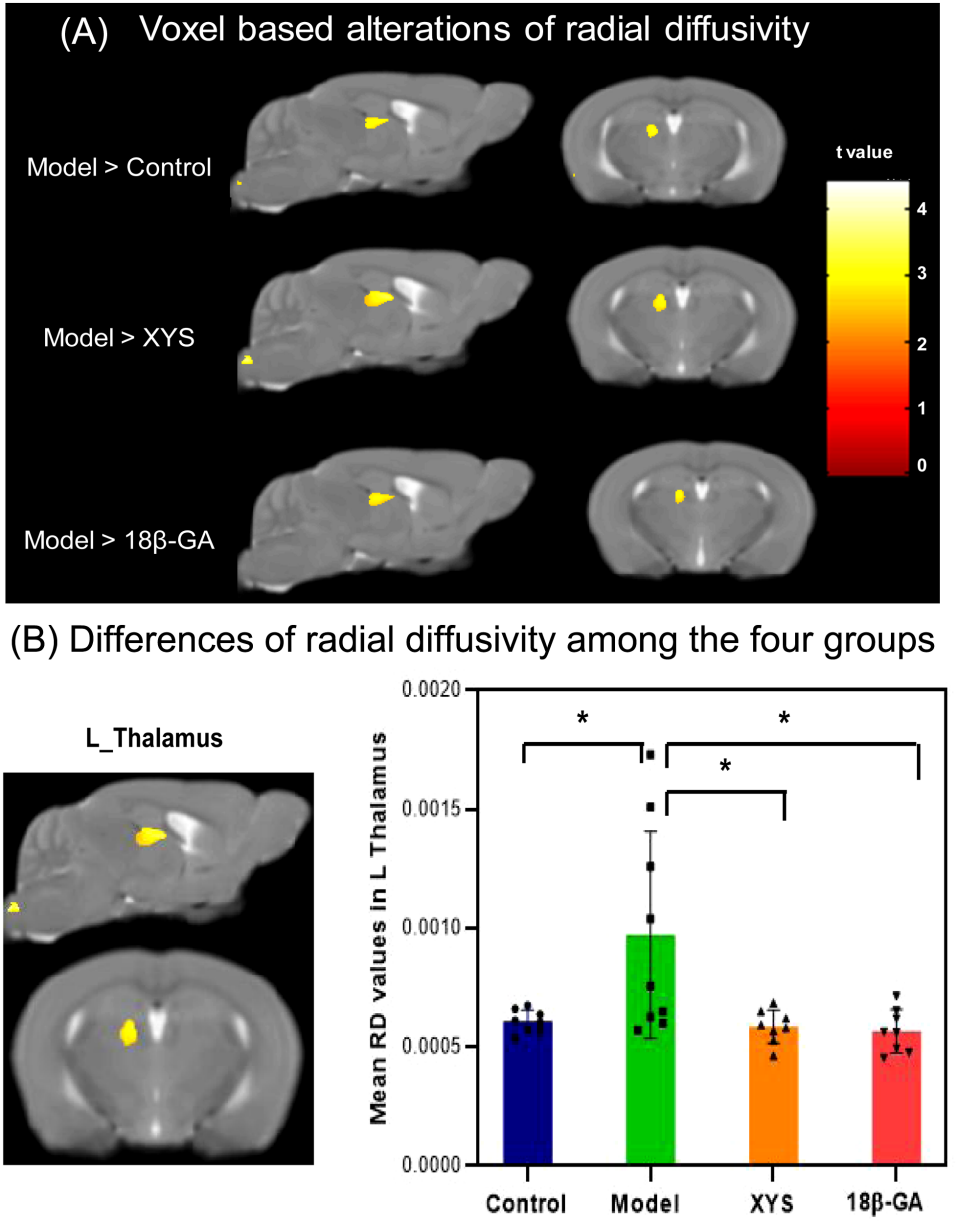
From RD maps, WM microstructural differences were detected among the four groups. **(A)** The model mouse group showed a significant increase in RD compared with the other three groups by voxel-based statistics. **(B)** Effect of the XYS treatment on brain diffusion properties of RD in the left thalamus between groups. post t-test: *p<0.05.

Voxel-based analyses of RD maps were performed and are illustrated in **Figure 6** and **Table 1**. The CSDS model group exhibited significantly higher RD values than the other three groups in left TH (XYS treated: t = 2.394, p = 0.031; 18β-GA treated: t = 2.53, p = 0.024).

### 3.4 SVM classification results

**Figure 7:**
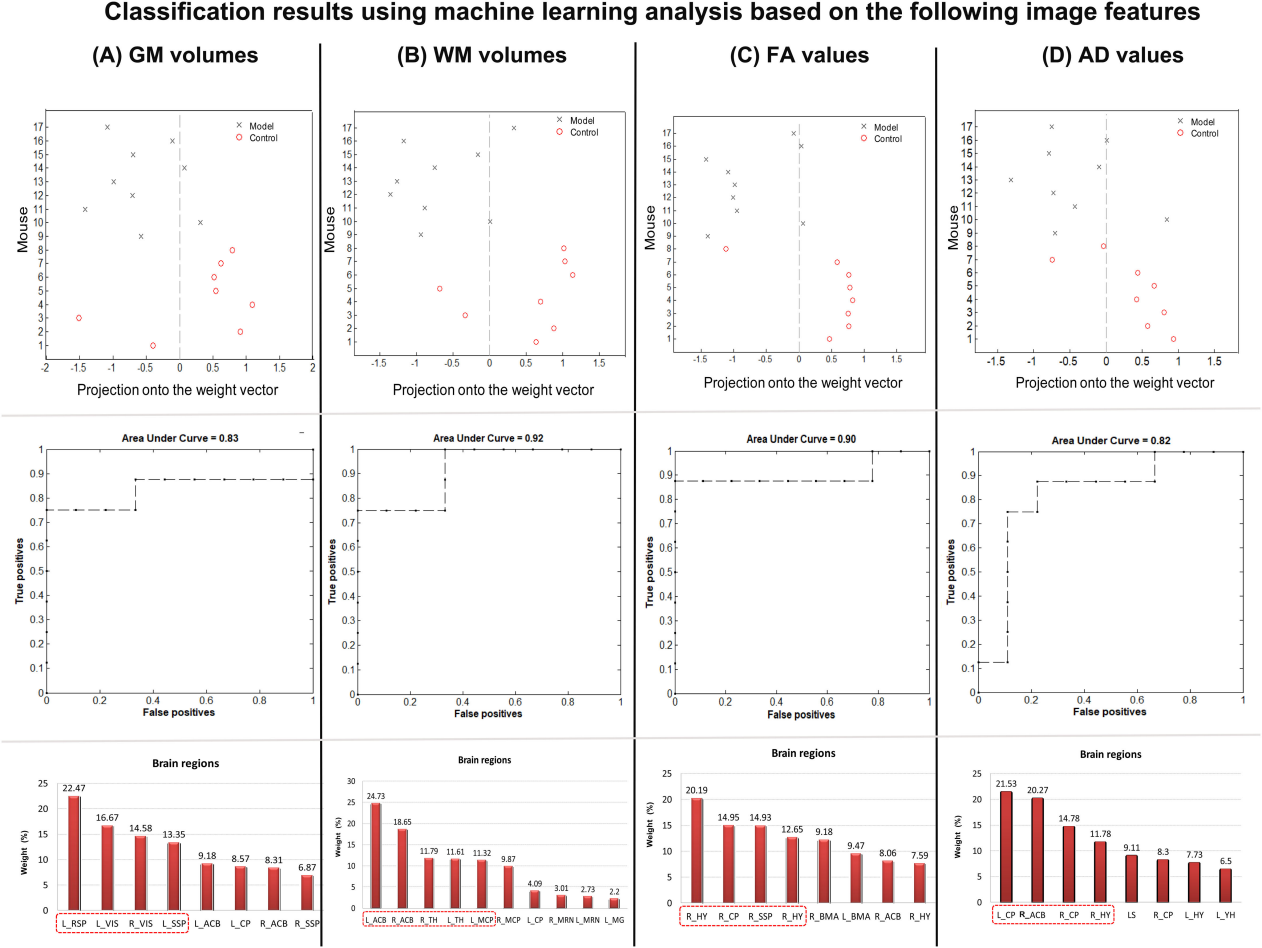
Classification results using machine learning analysis based on **(A)** GM volumes, **(B)** WM volumes, **(C)** FA values, and **(D)** AD values. The up line showed the prediction values per mouse of the classification model. The middle line shows the area under curve values in each classification model. The down line shows the contribution of each interesting area for the classification.

The classification results based on GM volume information are presented in **Figure 7A**. Regions exhibiting significant changes in GM volume (as shown in **Figure 2A**) were selected as a mask, and the GM volume information was regarded as a feature in the classification model. The SVM classification model achieved an overall accuracy of 76.39%, which demonstrated statistical significance at p < 0.041. The model demonstrated a sensitivity of 75% and specificity of 77.78%. The AUC of the classification model was determined to be 0.83. Regarding GM volume, the most informative region for distinguishing between CSDS model mice and control mice included left RSP, bilateral VIS, and left SSP.

Additionally, the classification results based on WM volume information are presented in **Figure 7B**. The regions exhibiting significant changes in WM volume (as shown in **Figure 2B**) were selected as a mask, and the WM volume information was regarded as a feature in the classification model. The SVM classification model achieved an overall accuracy of 76.39%, which demonstrated statistical significance at p < 0.037. The model demonstrated a sensitivity of 75% and specificity of 77.78%. The AUC of the classification model was determined to be 0.92. Regarding WM volume, the most informative region for distinguishing between CSDS model mice and control mice included bilateral ACB, bilateral TH, and left MCP.

The classification results based on DTI FA values are presented in **Figure 7C**. A mask was created by selecting all the regions that exhibited significant changes in FA (as shown in **Figure 4A**), and the FA information from these regions was considered as a feature in the classification model. The SVM classification model achieved an overall accuracy of 82.64%, which demonstrated statistical significance at p < 0.007. Furthermore, the model demonstrated a sensitivity of 87.5% and a specificity of 77.78%. The AUC value for the classification model was determined to be 0.9. Notably, among the FA values, bilateral HY, right CP, and right SSP emerged as the most informative region for distinguishing between CSDS model mice and control mice.

The classification results based on DTI AD values are presented in **Figure 7D**. Regions exhibiting significant changes in FA (as showing in **Figure 5A**) were selected as a mask, and the AD information was utilized as a feature in the classification model. The SVM classification model achieved an overall accuracy of 76.39%, which demonstrated statistical significance at p < 0.039. Moreover, the model exhibited a sensitivity of 75% and specificity of 77.78%. Notably, the AUC of the classification model reached 0.82. Regarding AD values, the bilateral CP, right HY, and ACB emerged as the most informative region for distinguishing between CSDS model mice and control mice.

## 4 Discussion

This investigation aimed to determine the potential brain structural plasticity induced by XYS therapy in a mouse model of CSDS, and whether the observed changes in GM and WM structure can reflect the antidepressant-like effect of XYS. To achieve this objective, we utilized CSDS model mice and acquired T2-weighted images and DTI data. By comparing voxel-wise alterations in brain volume and diffusion metrics with control mice, significant structural changes were found in regions including the ACB, SSP, TH, HY, BMA, CP, and RSP. Notably, both XYS pretreatment and 18β-GA pretreatment prevented disruptions in brain volume and diffusion metrics within certain regions mentioned above, such as bilateral HY, right SSP, right ACB, bilateral CP, and left TH. Furthermore, a classification model based on the neuroimaging features from the right ACB, right HY, and right CP demonstrated high accuracy for distinguishing between CSDS model mice and controls.

### 4.1 Significant decrease of the brain structure in mouse after chronic stress

Animal models serve as indispensable tools for investigating human psychiatric disorders. It is well established that a significant portion of our current understanding regarding the underlying mechanisms of depression has been derived from animal models. Among these, the CSDS is frequently employed to induce depression-like behaviors in mice, such as avoidance and anhedonia (28, 29, 36). In this study, CSDS model mice exhibited a notable reduction in brain volume and altered diffusion indices (decreased FA and increased AD) specifically within the ACB and CP). The ACB and CP are key structures implicated in various aspects of reward processing (37). Previous studies have demonstrated their role as central hubs within the brain’s reward pathways involved in mood regulation and emotion processing (38, 39). Prior studies utilizing T1-weighted structural MRI studies consistently reported volumetric reductions within the ACB in major depressive disorder patients (40, 41). Additionally, functional MRI investigations revealed significantly attenuated responses within the left ACB and bilateral caudate among individuals with major depression compared with healthy controls (42). Furthermore, alterations in neuroimaging measures such as volume and activity within the ACB and caudate were found to be associated with severity of anhedonia symptoms and overall depression severity among depressed patients (40, 42). Consistent structural changes observed across these regions may suggest that dysfunction within the ACB and CP could be key factors associated with dysfunction in the mouse model of depression-like behavior induced by CSDS. Our machine learning results also support this notion. We utilized various types of neuroimaging features for machine learning analysis. We found that both brain volumes and diffusion features achieved high accuracy in classifying CSDS model mice from controls. Furthermore, we identified significant contributions to classification from the right ACB and right CP, further confirming their crucial role in depression pathology. The structural deficits observed in these regions also contribute to the neuropathology of depression.

The amygdala, a limbic system structure in emotion and motivation processing, has shown conflicting results regarding volume changes (increase or decrease or no change) in patients with major depression (43–45). Animal studies have indicated that chronic stress generally leads to increased spine density in the amygdala (46). In contrast, a previous study on mouse found that chronic restraint stress caused a decrease specifically in the medial amygdala (47). In our present study, we also assessed amygdala volume but did not observe any significant differences between CSDS mice and controls. However, when examining brain microstructural changes, we discovered a significant decrease in FA values within the bilateral BMA of CSDS mice compared with normal controls. These structural changes likely involve alterations at the circuit, cellular, and/or synaptic level (48, 49). Recent studies using a chronic unpredictable mild stress mouse model of depression have reported significant decreases in brain activity within the BMA region (24, 25). It is plausible that these alterations in brain activity may impact brain microstructure and subsequently affect water diffusion properties within this region. To validate this hypothesis further, future studies should incorporate functional MRI data to investigate the functional connectivity between regions exhibiting significant changes of their diffusion indices.

The thalamus is considered as a complex sensory information node that control motion, memory, and arousal. A previous study has demonstrated the involvement of this region in both emotional salience network and the emotional regulation network (50). In the present study, we observed a significant decrease of the volumes in bilateral TH and significant changes of the diffusion indices (decreased AD and increased RD) in the left TH. These findings are consistent with a previous clinical investigation, which reported significant volume reduction and shape alterations in the left TH among patients with depression, where these structural changes were significantly correlated with depression severity (51). Tractography analysis of the anterior limb of the internal capsule revealed extensive projections to various regions including the entire thalamus, hypothalamus, and brainstem, all of which have been implicated in depressive syndrome (52). The present results suggest that abnormal water diffusion patterns and structural disruptions within the thalamus may contribute to aberrant information flow between the limbic system and thalamus. Emotional dysregulation has been consistently observed among individuals with depression. Additionally, we found significant decreases in diffusion indices (FA and AD values) within HY in our CSDS mouse model. A previous study has highlighted various nuclei within HY as crucial hubs within stress-related neurocircuit (53). Early review study by Schindler et al. (54) has reported either reduced or normal volumes of HY among individuals with depression. Although no significant volume changes were detected for HY in our CSDS mice model, we did observe substantial reductions in FA and AD values within this structure compared with control mice. The changes in diffusion metrics observed in this study can be attributed to a reduction in the total number of neurons in the HY of the model mice (55). Although there was no significant change in volume, a decrease in neuron number resulted in decreased neuronal density and enhanced lateral diffusion ability of water molecules within the HY. Correspondingly, AD and FA values were found to be decreased specifically in the CSDS mouse model.

In addition to subcortical regions, structural alterations were also detected within cortical regions (such as the SSP and RSP) of CSDS mice. These findings suggest that impairments within cortical–subcortical pathways may contribute to the pathology observed in these regions, highlighting their potential role as a pathophysiological target for depression (56). Furthermore, our results support previous studies indicating widespread brain structural abnormalities among patients with depression (15), which correspond to alterations within brain functional network regions. The brain structural change can be found at the whole brain. Thus, the significant changes of brain volume and diffusion indices were simultaneously detected of the CSDS mouse in the present study.

### 4.2 XYS-related modulation of brain structure in CSDS model mouse

XYS is a classic traditional Chinese herbal medicine, which has been widely used to cure the patients with mental disorders in China for centuries. Previous clinical and animal studies have established the safety and efficacy of this medicine in the therapy of depressive disorder (7, 25). The behavioral findings from our present study also demonstrate that XYS exhibits prophylactic antidepressant-like effects, comparable with those of fluoxetine (30). Therefore, it can be concluded that XYS demonstrates prophylactic antidepressant-like effects in mice exposed to CSDS. However, few studies have investigated the alternations in whole brain structure following treatment with XYS in CSDS mice. Our study reveals an increased tendency towards brain volume and diffusion indices (FA values) within the disrupted regions of CSDS model mice treated with XYS. Notably, compared with the CSDS model group, significant increases were observed in FA values within the right HY, right ACB, and right SSP of the XYS treatment group. Additionally, we observed suppression of significantly increased RD within the left TH region in the XYS treatment group. Importantly, all these regions exhibiting antidepressant effects are associated with emotion or reward-related circuits. Previous studies have confirmed a correlation between structural covariance of ACB and illness duration (57), making it a target for deep brain stimulation as a potential treatment for resistant depression (38). Furthermore, TH is considered as a crucial causal hub for depression and may serve as a downstream target for depression treatment (50). All these findings support the notion that the therapeutic effect in depression disorder is associated with regional structure recovery of the brain. As anticipated, prophylactic treatment with XYS can prevent stress-induced structural alterations in ACB, TH, HY, and SSP. All these regions are key regions involved in stress response and recovery through XYS treatment. Similar neuroimaging improvements were also observed in the 18β-GA treatment group, consistent with our expression. This could be attributed to 18β-GA being identified as the primary active component in the XYS formulation according to our recent study (30). The observed structural recovery in ACB, TH, HY, and SSP may represent a neuroimaging manifestation of 18β-GA exerting its antidepressant-like actions in the CSDS model. Another possible explanation for these imaging changes could be based on myelination theory. Myelin degradation via ubiquitination is closely linked to astrocytes and microglial activities that are implicated in depression pathology. Previous studies have demonstrated that alterations in myelin may underlie ketamine’s long-lasting antidepressant effects (58, 59). Reduced myelination has been associated with depression pathology as well. Consistent with ketamine’s anesthetic effect, our recent study also revealed rapid and enduring antidepressant-like effects of XYS and 18β-GA administration in CSDS-susceptible mice (30). Therefore, significant changes observed in diffusion indicators within this study can also be explained by myelination theory. Myelin degradation was detected within the model group whereas reduced myelination was restored following treatment intervention groups. Why are the prophylactic antidepressant-like effects of these two treatments primarily observed in specific regions rather than others? To address this question, we integrated the machine learning findings from our study. Our results indicate that these regions contribute significantly to the classification, suggesting their susceptibility to depression induced by CSDS. The therapeutic efficacy of XYS and 18β-GA may be attributed to their ability to prevent brain structure disruption in these susceptible regions.

While both treatment groups exhibited similar neuroimaging expressions (structural recovery in ACB, TH, HY, and SSP) under the prophylactic effects, there were differences in change patterns between the two groups for certain regions. For instance, the XYS treatment group demonstrated significant improvement in brain volume specifically within the left SSP and left RSP, whereas such changes were not significant in the 18β-GA treatment group. Additionally, in the XYS-treated group, diffusion indices (FA and AD) significantly increased in right CP and right ACB but showed no significant change in the 18β-GA-treated group. Significantly enhanced AD values in left TH and left HY were detected only in the 18β-GA-treated group. These findings are intriguing and important as they suggest that differential effects on brain structure between XYS and 18β-GA could be attributed to variations in their composition. Specifically, XYS is prepared using a traditional recipe consisting of eight commonly used Chinese herbs with multiple targets at different levels according to previous studies (6, 7). According to the synaptic plasticity theory, depression is closely associated with downregulation of brain-derived neurotrophic factor (BDNF). The regulation of BDNF expression is considered as the main pathway for both 18β-GA and XYS therapies. In addition to the main compound 18β-GA in the brain following XYS injection, XYS has a more complex composition that includes unidentified compounds, which can also affect BDNF transcription via alternative pathways. Therefore, we observed significant enhancement of neuroimaging indices in certain regions after treatment with XYS but not with 18β-GA. Further experiments will be conducted to explore differences in treatment efficacy between XYS and 18β-GA in depression.

There are several limitations in this assessment of mouse structure changes due to XYS treatment. Firstly, the small sample size in this study has resulted in reduced statistical power for data analysis. Secondly, the resting-state functional MRI data were not collected, which hinders a comprehensive understanding of brain activity and functional connectivity underlying alterations in brain structure metrics among CSDS mice following treatment. Thirdly, only model-sensitive mice were included in the present study, whereas those that failed to be successfully modeled were disregarded. Future studies should encompass these unsuccessfully modeled mice to fully elucidate the mechanisms involved in this type of depression.

## 5 Conclusion

In the present study, we used modern neuroimaging tools to elucidate the underlying mechanisms of depression and investigate the prophylactic antidepressant-like effects of XYS. By combining voxel-based morphometry of T2-weighted images and voxel-based analysis of diffusion tensor image, significant alterations in brain volume and diffusion indices were observed in various regions including ACB, SSP, TH, HY, BMA, CP, and RSP in CSDS model mice. Classification models based on different types of neuroimaging features achieved high accuracy in distinguishing CSDS model mice from control mice. Notably, both XYS and 18β-GA treatments effectively prevented disruptions in brain volume and diffusion metrics across several regions such as bilateral HY, right SSP, right ACB, bilateral CP, and left TH. These findings suggest that prophylactic treatment with XYS modulate brain macro- and microstructural changes within specific regions to exert antidepressant-like effects. Additionally, our results highlight the significant contribution of 18β-GA to the antidepressant-like effects of XYS in mice. Furthermore, our discovery underscores how multimodal neuroimaging techniques can provide complementary and redundant information to enhance our understanding of the antidepressant-like effects exhibited by XYS.

## Data Availability

The raw data supporting the conclusions of this article will be made available by the authors, without undue reservation.
